# Supplementary material for: Carotid Atherosclerosis Progression in Postmenopausal Women Receiving a Mixed Phytoestrogen Regimen: Plausible Parallels with Kronos Early Estrogen Replacement Study
Source: Biology (Basel). 2020 Mar 6;9(3):48. doi: 10.3390/biology9030048 (PMC7150954; doi:10.3390/biology9030048)
Supplement: Supplementary file 1 [file biology-09-00048-s001.zip › biology-709792-supplementary/Figure S1.doc]

**CONSORT Flow Diagram**

***Early postmenopausal group***

**Allocation**

**Analysis**

**Follow-Up**

**Enrollment**

Assessed for eligibility (n=374)

Excluded (n=59)

♦  Not meeting inclusion criteria (n=53)

♦  Declined to participate (n=6)

♦  Other reasons (n=0)

Analysed (n=155)
♦ Excluded from analysis (give reasons) (n=0)

Lost to follow-up (due to non-complience of preparation administration and non-attendance of the intermediate visit) (n=6)

Discontinued intervention (give reasons) (n=0)

Allocated to intervention (n=161)

♦ Received allocated intervention (n=161)

♦ Did not receive allocated intervention (give reasons) (n=0)

Lost to follow-up (due to non-complience of preparation administration and non-attendance of the intermediate visit) (n=3)

Discontinued intervention (give reasons) (n=0)

Allocated to intervention (n=154)

♦ Received allocated intervention (n=154)

♦ Did not receive allocated intervention (give reasons) (n=0)

Analysed (n=151)
♦ Excluded from analysis (give reasons) (n=0)

Randomized (n=315)

**Phytoestrogen subgroup**

**Placebo subgroup**
