# Supplementary material for: Carotid Atherosclerosis Progression in Postmenopausal Women Receiving a Mixed Phytoestrogen Regimen: Plausible Parallels with Kronos Early Estrogen Replacement Study
Source: Biology (Basel). 2020 Mar 6;9(3):48. doi: 10.3390/biology9030048 (PMC7150954; doi:10.3390/biology9030048)
Supplement: Supplementary file 1 [file biology-09-00048-s001.zip › biology-709792-supplementary/Figure S2.doc]

**CONSORT Flow Diagram**

**Late postmenopausal group**

**Allocation**

**Analysis**

**Follow-Up**

**Enrollment**

Assessed for eligibility (n=267)

Excluded (n=27)

♦  Not meeting inclusion criteria (n=27)

♦  Declined to participate (n=0)

♦  Other reasons (n=0)

Analysed (n=119)
♦ Excluded from analysis (give reasons) (n=0)

Lost to follow-up (give reasons) (n=0)

Discontinued intervention (give reasons) (n=0)

Allocated to intervention (n=120)

♦ Received allocated intervention (n=119)

♦ Did not receive allocated intervention (due to technical reasons) (n=0)

Lost to follow-up (give reasons) (n=0)

Discontinued intervention (give reasons) (n=0)

Allocated to intervention (n=120)

♦ Received allocated intervention (n=112)

♦ Did not receive allocated intervention (due to technical reasons) (n=0)

Analysed (n=112)
♦ Excluded from analysis (give reasons) (n=0)

Randomized (n=240)

**Phytoestrogen subgroup**

**Placebo subgroup**
